# Roflumilast Prevents the Metabolic Effects of Bleomycin-Induced Fibrosis in a Murine Model

**DOI:** 10.1371/journal.pone.0133453

**Published:** 2015-07-20

**Authors:** Javier Milara, Esteban Morcillo, Daniel Monleon, Herman Tenor, Julio Cortijo

**Affiliations:** 1 Clinical Research Unit, University General Hospital Consortium, Valencia, Spain; 2 CIBERES, Health Institute Carlos III, Valencia, Spain; 3 Research Foundation of General Hospital of Valencia, Av. tres cruces s/n., E-46014, Valencia, Spain; 4 Department of Pharmacology, Faculty of Medicine, University of Valencia, Valencia, Spain; 5 Fundacion Investigacion Hospital Clinico Universitario/INCLIVA, Valencia, Spain; French National Centre for Scientific Research, FRANCE

## Abstract

Fibrotic remodeling is a process common to chronic lung diseases such as chronic obstructive pulmonary disease (COPD), pulmonary fibrosis, acute respiratory distress syndrome and asthma. Based on preclinical studies phosphodiesterase 4 (PDE4) inhibitors may exhibit beneficial anti-inflammatory and anti-remodeling properties for the treatment of these respiratory disorders. Effects of PDE4 inhibitors on changes in the lung metabolome in models of pulmonary fibrotic remodeling have not yet been explored. This work studies the effects of the PDE4 inhibitor roflumilast on changes in the lung metabolome in the common murine model of bleomycin-induced lung fibrosis by nuclear magnetic resonance (NMR) metabolic profiling of intact lung tissue. Metabolic profiling reveals strong differences between fibrotic and non-fibrotic tissue. These differences include increases in proline, glycine, lactate, taurine, phosphocholine and total glutathione and decreases in global fatty acids. In parallel, there was a loss in plasma BH4. This profile suggests that bleomycin produces alterations in the oxidative equilibrium, a strong inflammatory response and activation of the collagen synthesis among others. Roflumilast prevented most of these metabolic effects associated to pulmonary fibrosis suggesting a favorable anti-fibrotic profile.

## Introduction

Pulmonary fibrotic remodeling is characterized by the development of excess fibrous connective tissue in the lungs. This process is common to lung diseases such as chronic obstructive pulmonary disease (COPD), pulmonary fibrosis, acute respiratory distress syndrome and asthma among others [1]. A persistent airway inflammation may support fibrotic remodeling in COPD or asthma. Therefore, in these latter ailments, anti-inflammatory treatment may also mitigate a risk of airway fibrotic remodeling. Among the variety of emerging strategies currently in development for diminishing inflammation in respiratory diseases, the inhibition of phosphodiesterase (PDE) 4 has shown promising results [2]. PDEs are enzymes which mediate the hydrolysis of cyclic adenosine or guanosine monophosphates [3]. While the distribution of PDEs is rather wide, inflammatory cells preferentially express PDE4 [4–6]. As a consequence, PDE4 inhibitors exhibit beneficial anti-inflammatory properties for the treatment of respiratory diseases, including asthma and COPD [7–9]. In fact, roflumilast is the first PDE4 inhibitor approved for COPD as a treatment to reduce the risk of exacerbations in patients with severe COPD associated with chronic bronchitis and a history of exacerbations. Besides the anti-inflammatory effects of roflumilast, considered critical towards its clinical benefits in COPD, results from additional *in vitro* and *in vivo* studies have shown that the PDE4 inhibitor curbs a broad spectrum of lung fibroblast functions such as myofibroblast transition and ECM generation or epithelial to mesenchymal transition (EMT) *in vitro* and mitigates bleomyin-induced lung fibrosis *in vivo* [2,10–12]. However, there is a need for further molecular studies in relevant models to better understand potential clinical implications.

Metabolomics is being described as a promising tool in biomedical research with interesting applications in medical research and clinical environments. Metabolomics permits a quantitative measurement of the multiparametric metabolic response of living systems to pathophysiological stimuli by simultaneously examining dynamic changes in hundreds of low-molecular-weight metabolites in tissues or biofluids [13]. The use of metabolomics to study inflammatory lung diseases is quite recent, with an emphasis in its potential for the discovery of biomarkers that characterize these diseases as well as in defining different disease phenotypes [14]. In fact, most of the metabolomics-related literature is focused on the use of this technique for the early diagnosis and discovery of biomarkers in diseases, such as asthma, cystic fibrosis and COPD [15]. However, less is known about metabolomics and lung fibrosis. In this regard, recent metabolomic investigations identified an over-expression of the lactic acid metabolite in lung tissues of idiopathic pulmonary fibrosis patients, supporting a concept that acidification of lung tissue activates the latent form of transforming growth factor beta (TGFβ) and the subsequent myofibroblast differentiation and invasion [16]. Thus, based on metabolomic data, an ideal anti-fibrotic drug should improve the metabolomic profile characteristic of pulmonary fibrotic remodeling.

This work aims to characterize the metabolic profile of the well characterized and commonly used mouse model of bleomycin-induced lung fibrosis that in its fibrotic phase partly mimics aspects of idiopathic pulmonary fibrosis [17]. In addition, the effect of the PDE4 inhibitor roflumilast that was previously shown by us to mitigate bleomycin induced lung fibrosis [10] on this metabolic profile was explored. To this end, we used nuclear magnetic resonance (NMR) metabolomics of intact lung tissue and multivariate analysis for the detection of global metabolic changes and patterns associated to both, bleomycin-induced lung-injury and its prevention by roflumilast.

## Material and Methods

### Animals

Animal experiments were performed in accordance with the European Community and Spanish regulations for the use of experimental animals and approved by the institutional committee of animal research. The murine model used specific pathogen-free male C57Bl/6J mice (Charles River, Barcelona, Spain) at 8 weeks of age which are reported to mount a robust early inflammatory response followed by pulmonary fibrotic remodeling secondary to bleomycin [10]. Mice were housed with free access to water and food under standard conditions: relative humidity 55 ± 10%; temperature 22 ± 3°C; 15 air cycles/ per hour; 12/12 h Light/Dark cycle.

### Experimental design

The murine model of blemycin-induced lung fibrosis was performed as previously described [10]. Briefly, mice were anaesthetized with ketamine/medetomidine and then instilled on day 1 with a single intratracheal dose of 3.75 U/kg bleomycin (dissolved in 50 μL of saline). This dose of bleomycin reproducibly generated pulmonary fibrosis in previous experiments. Sham-treated mice received an identical volume of intratracheal saline instead of bleomycin. In this mice model, bleomycin triggers an initial inflammatory lung response from day 1 through day 7 followed by a fibrotic response beyond day 7 (>7 days after bleomycin) [10]. The PDE4 inhibitor roflumilast was administered in a *preventive protocol*, to measure metabolic changes related with inflammation and fibrosis, which consisted in animals receiving roflumilast once daily, starting from the day of bleomycin administration (day 1) until the end of the experiment at day 14. Mice were allocated to the following groups: (i) saline + vehicle (17 animals); (ii) bleomycin + vehicle (14 animals); (iii) bleomycin + roflumilast 1 mg/kg/day (8 animals); and (iv) bleomycin + roflumilast 5 mg/kg/day, (4 animals). Roflumilast was given in methocel suspensions, once daily, p.o. by gavage in a volume of 10 mL/kg. At the end of the treatment period, mice were sacrificed by a lethal injection of sodium pentobarbital followed by exsanguination. After opening the thoracic cavity, trachea and lungs were removed *en bloc*. Lungs were then processed for histological and metabolomic studies and blood was collected to determine plasma tetrahydrobiopterin (BH4), and dihydrobiopterin (BH2). Lung tissue samples were collected from whole left lung that was homogenized to avoid variability of typical located fibrotic areas.

### Histology

Lung histology was conducted as previously reported [18]. Tissue blocks (4 mm thickness) were stained with Masson’s trichrome ((Sigma-Aldrich, Madrid, Spain) to detect fibrosis and collagen deposition. Severity of lung fibrosis was scored on a scale from 0 (normal lung) to 8 (total fibrotic obliteration of fields) according to Ashcroft [19].

### Determination of BH4/BH2

To measure plasma BH4 and BH2 venous blood was collected in EDTA tubes containing 0.1% (w/v) dithioerythritol (DTE) as antioxidant. Total biopterin equals the combined sum of BH2, BH4 and fully oxidized biopterin. Differential oxidation mediated by iodine permits the measurement of BH4 concentration. Under acidic conditions BH4 and BH2 are oxidized to biopterin, while under basic conditions only BH2 is oxidized to biopterin, and BH4 undergoes side-chain cleavage to form pterin. The difference in biopterin content between the two oxidations represents the actual BH4 levels [20]. In brief, the acidic oxidation was realized as follows: 90 μl of plasma and 10 μl of the internal standard (rhamnopterin 400 nM) were acidified by the addition of 20 μl HCl (1M) and 50 μl of I2/IK solution (1% (w/v) iodine in 2% (w/v) potassium iodide). Samples were mixed and incubated for 1 h in the dark at room temperature. The reaction was stopped adding 10 μl of 5% (w/v) ascorbic acid and 20 μl of water. The basic condition was identical to the preparation of the acidic condition with the exception that HCl was substituted by the addition of 20 μl NaOH (1M). Samples were added, mixed and incubated for 1 h in obscurity at room temperature followed by the addition of 10 μl of 5% (w/v) ascorbic acid and 20 μl HCl (2M).

The HLPC system consisted of a Shimadzu LC-10ADvp isocratic pump, Shimadzu SIL-10ADvp auto-injector, Shimadzu RF10Avp fluorescence detector and Shimadzu SCL-10Avp controller. HPLC system control and data processing were performed by Shimadzu LCM Solutions software (Shimadzu, Tokyo, JP). The analytical method was validated in our laboratory in accordance to FDA Guidance for Industry [21]. The separation was performed as described by Fiege et al [22]. The stationary phase included precolumn and column Sherisorb ODS1 5μm (4.6 x 250 mm; Waters Barcelona, Spain), and a mobile phase consisting in 1.5 mmol/L potassium hydrogen phosphate at pH 4.6 with 10% methanol, at a flow rate of 1 mL/min at room temperature. The fluorescence detector was set at 350nm (excitation) and 450nm (emission). Prior to inject into the HPLC system, samples were filtered through a 10000 MW microfilter (Multiscreen, Millipore) at 3000g for 1h at 10°C for physical desproteinisation. Quantification of BH4 and BH2 was made by interpolation to a standard curve of biopterin (1, 5, 10, 25, 50, 75 and 100 ng/ml).

### NMR spectroscopy

NMR HR-MAS spectra were obtained for 43 samples of lung tissue (10–20 mg). Total sample preparation time for each sample prior to NMR detection was less than 5 min. All the material to be in contact with the tissue was pre-cooled to reduce tissue degradation during the sample preparation process. Frozen samples were taken from the ultra-freezer and immediately placed in a cryo-vial and in liquid N_2_ until insertion in a 4-mm outer diameter ZrO_2_ rotor. The HR-MAS tissue sample was split from the whole frozen mass submerged in liquid nitrogen. The pre-cooled rotor was filled with cooled D_2_O after tissue sample insertion. Cylindrical inserts were used in all the cases, limiting the rotor inner volume to 50 μl. Exceeding D_2_O was removed before rotor sealing. Tissue samples were weighted in the rotor before D_2_O addition and HR-MAS measurements. Tissue fragments were weighted exclusively for sample preparation purposes. The mean sample weight was 25 ± 8 mg.

All spectra were recorded in a Bruker Avance DRX 600 spectrometer (Bruker GmbH, Rheinstetten, Germany) operating at a ^1^H frequency of 600.13 MHz. The instrument was equipped with a 4 mm triple resonance ^1^H/^13^C/^15^N HR-MAS probe with magnetic field gradients aligned with the magic angle axis. For all experiments, samples were spun at 5000 Hz to keep the rotation sidebands out of the acquisition window. Lock homogeneity was achieved by extensive coil-shimming using the 1D water pre-saturation experiments in interactive mode as control. Alanine doublet at 1.475 ppm was used for lock homogeneity shimming and for chemical shift referencing, as described elsewhere [23]. Nominal temperature of the sample receptacle was kept at 273K, using the cooling of the inlet gas pressures responsible for the sample spinning. This value corresponded to the temperature measured from the thermocouple just below the rotor in the probe. The effect of sample rotation was to slightly increase this value. Internal measurement using a 100% MeOH sample in a 4 mm rotor spinning at the same frequency provided a corrected internal value of 277K. In order to minimize the effects of tissue degradation, which would alter the metabolite composition of the biopsy, all spectra were acquired at this temperature of 277K. A total of 10 min was allowed for the temperature of the sample to reach steady state before spectra were acquired. A single-pulse pre-saturation experiment was acquired in all the samples. Number of transients was 256 collected into 32 k data points for all the experiments. Water pre-saturation was used during 1 second along the recycling delay for solvent signal suppression. Spectral widths were 8000 Hz for 1h. Before Fourier transformation, the free induction decay was multiplied with a 0.3 Hz exponential line broadening. For assignment purposes, two-dimensional (2D) homo (2D-TOCSY) and heteronuclear (2D-1H, 13C-HSQC) experiments were acquired on selected samples.

### Data analysis

All 43 spectra were processed using MNova 5.3 (MestreLab S.A., Santiago de Compostela, Spain) and transferred to MATLAB (MathWorks Inc, 2006) using in-house scripts for data analysis. All multivariate analysis was performed using the PLS_Toolbox library. The chemical shift region including resonances between 0.50 and 4.60 ppm and between 5.20 and 10.50 ppm was investigated. For comparison of global metabolic profiles from different animal protocols, the spectra were binned into 0.01 ppm buckets, normalized to total spectral integral and subsequently analyzed by Principal Components Analysis (PCA). The spectral binning and normalization minimized the impact of differences in tissue weight and cell content for the different samples. We cross validated our multivariate models by performing 10 technical replicates by choosing random training (30 samples) and validation (13 samples) data subsets. Cross-validation is a technique for assessing how the results of a statistical analysis will generalize to an independent data set.

Spectral signal integration by peak-fitting algorithms over assigned resonances provided relative levels of the corresponding metabolites. Only those signals with peak-fitting residual error lower than 10% were used in the study. Results are given as means and standard error. Statistical analysis of data was carried out by analysis of variance (ANOVA) followed by post hoc tests including Bonferroni’s correction as appropriate.

## Results

### Metabolic profiling quality control

**Fig 1:**
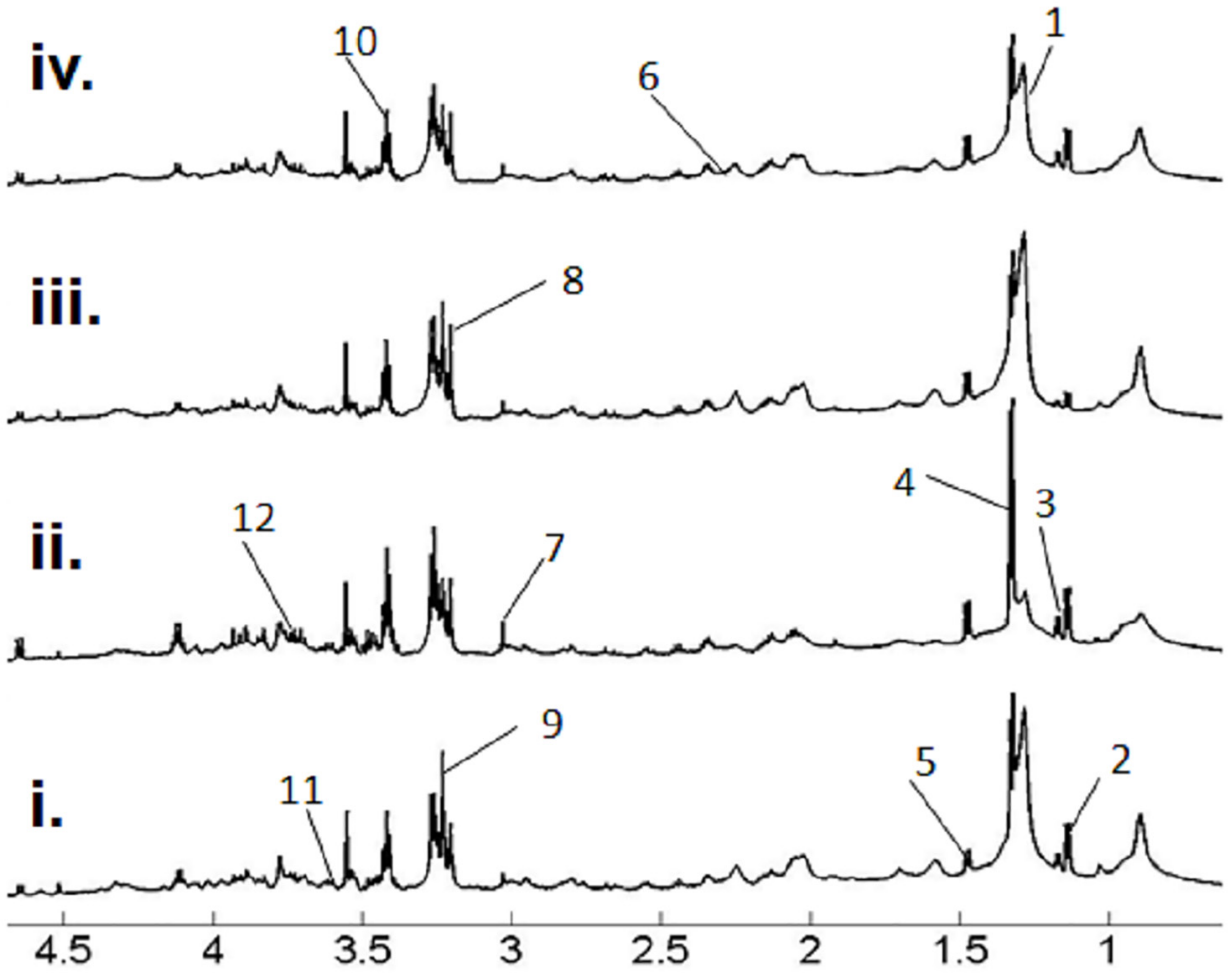
Representative NMR spectra (panels i, ii, iii, iv) for murine lung control tissue (panel i), lung tissue from animals treated with bleomycin + vehicle (panel ii), lung tissue from animals treated with bleomycin + roflumilast 1 mg/kg/day (panel iii) and lung tissue from animals treated with bleomycin + roflumilast 5 mg/kg/day (panel iv). Resonances belonging to most relevant metabolites have been labeled in the spectra (1: Fatty acids, 2: Leucine, 3:Isoleucine, 4:Lactate, 5:Alanine, 6:Total glutathione, 7:Creatine, 8:Choline, 9:Phosphocholine, 10:Taurine, 11:Glycine, 12:Glucose).

Mean ^1^H NMR spectra of lung tissue from the different animal protocols assayed are shown in Fig 1. All NMR spectra showed narrow line widths and adequate signal-to-noise ratios with well resolved spin-spin multiplicities. Metabolite spin systems and resonances were identified by using literature data [24] and additional 2D homo and multi-nuclear experiments collected in selected samples. The 1D single-pulse pre-saturation experiment provides complete and unambiguous identification of the metabolic pattern characterizing the examined tissues. Most relevant metabolites are labeled in Fig 1. In all spectra, the aliphatic region had prominent signals of water-soluble metabolites such as lactate, creatine, taurine, phosphoethanolamine, phosphocholine, and choline. The endogenous compounds detected in the spectra also include standard amino acids like glycine, proline, leucine, isoleucine, valine, alanine, lysine, asparagine, aspartic acid, glutamine and glutamate. Other signals observed included total glutathione, glucose, and glycerol. Prominent broad signals belonging to lipids and fatty acid chains were also detected. Principal component analysis (PCA) of processed NMR data was used to observe the overall variation in the data and spontaneous sample groupings.

**Fig 2:**
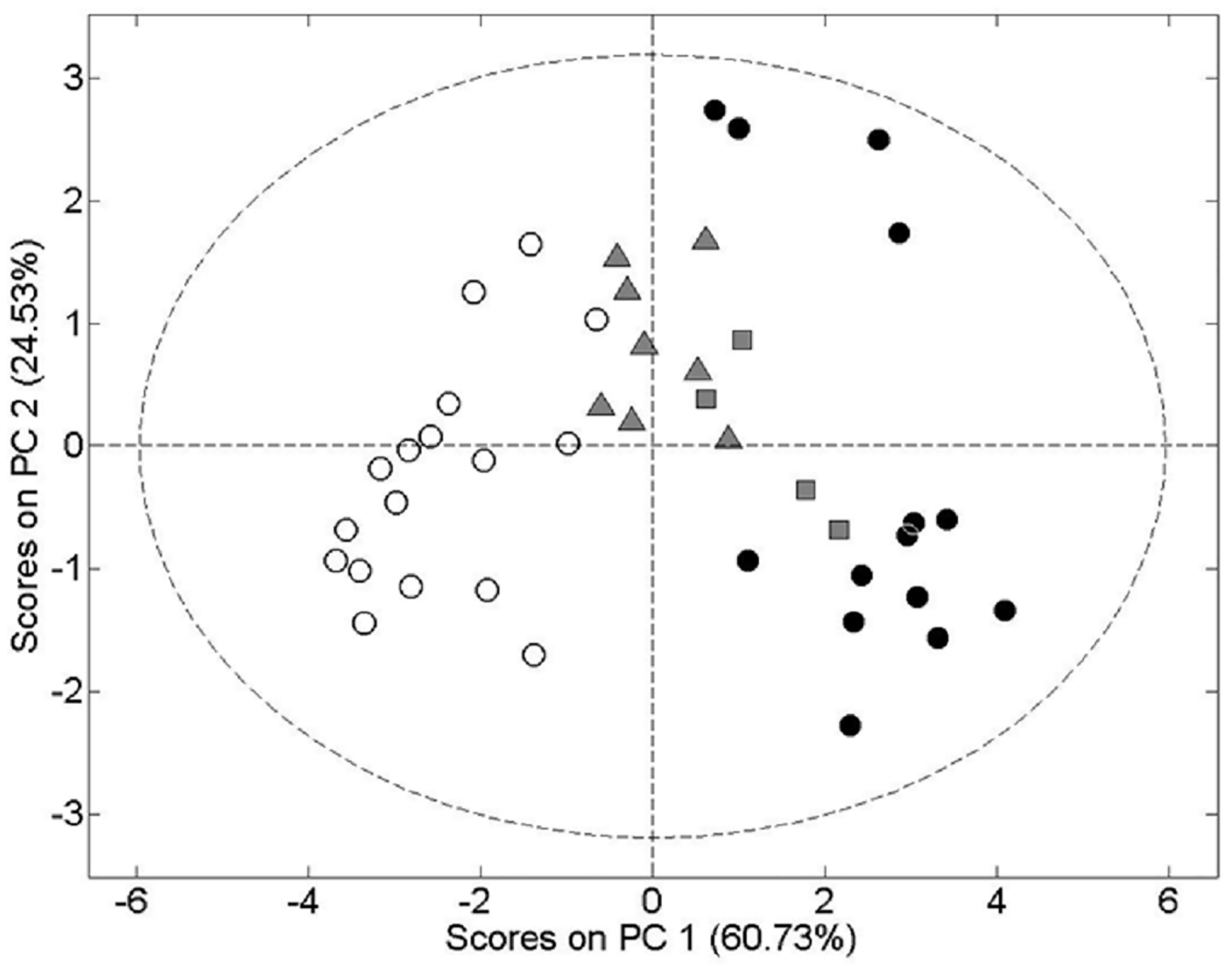
Scores plot of Principal Component (PC) Analysis to compare the metabolome of murine lung control tissue (white circles), lung tissue from animals treated with bleomycin + vehicle (black circles), lung tissue from animals treated with bleomycin + roflumilast at 1 mg/kg/day (gray triangles) and lung tissue from animals treated with bleomycin + roflumilast at 5 mg/kg/day (gray squares). PC1 and PC2 represent the two principal component analysis used to group individuals according with the amount of different metabolites in lung tissue.

Two principal components (PC) were calculated for the models with a total of 85% of variance being expressed (PC1, 60.73% + PC2, 24.53% = 85.23% (decimals are removed from the text). In this regard, metabolomic data was grouped in two groups PC1 and PC2 which explain the 85% of variability, thus creating a two dimensional figure in which each experimental group was located depending on the amounts (ppm) of different metabolites (Fig 2). No technical and biological potential outliers were detected in the scores plot of the PCA. Fig 2 showed the PCA scores plot on lung tissue samples spectra.

The PCA shows clear discrimination between control (white circles in Fig 2) and bleomycin treated mice (black circles in Fig 2). Interestingly, bleomycin + roflumilast (1 mg/kg/day; gray triangles in Fig 2) treated mice were closer to control mice than to bleomycin treated mice. Similar results were observed for the bleomycin + roflumilast at 5 mg/kg/day (gray squares in Fig 2) treated mice, although with more variability as a consequence of the small size of this experimental group. The distribution of the different animal protocols assayed, suggest that roflumilast prevents the metabolic effects of bleomycin-induced lung injury.

### Metabolic impact of bleomycin and roflumilast in lung tissue metabolic profile

**Fig 3:**
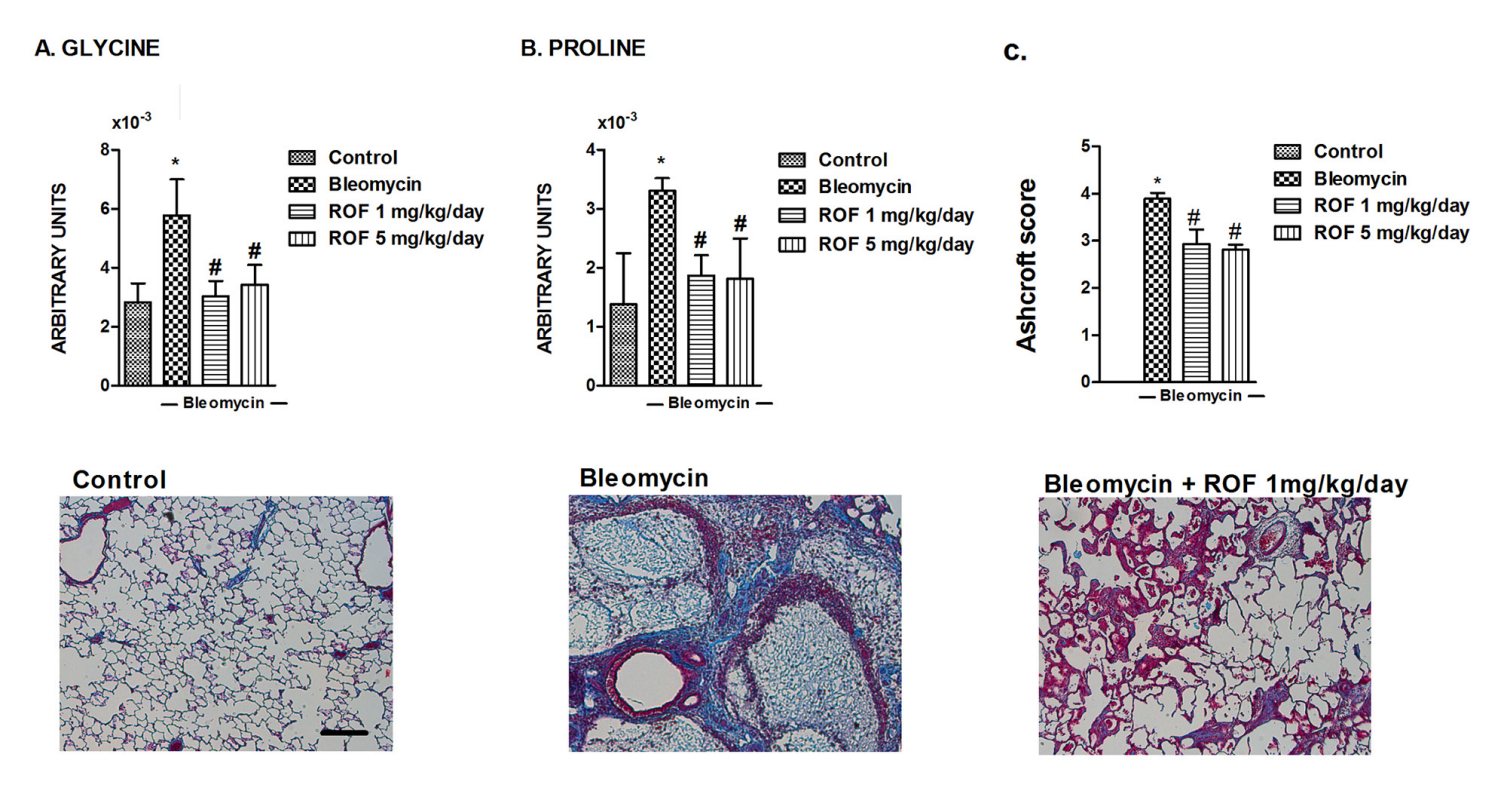
Effects of roflumilast on bleomycin-induced metabolites related to lung collagen deposition in mouse. Mice received a single dose of bleomycin (3.75 U/kg) intratracheally at day 1 and roflumilast (1 or 5 mg/kg/day p.o., once daily) or vehicle was administered from day 1 to 14 (*preventive protocol*) until analysis at day 14. Glycine (A) and proline (B) metabolites were assessed by NMR in murine lung control tissue (n = 17), lung tissue from animals treated with bleomycin + vehicle (n = 14), lung tissue from animals treated with bleomycin + roflumilast at 1 mg/kg/day (n = 8) and lung tissue from animals treated with bleomycin + roflumilast at 5 mg/kg/day (n = 4). In parallel experiments (C) lung sections were stained with Masson’s trichrome (lower panels, scale bar: 200μm; collagen stained in blue; Fibrosis Ashcroft scores were assessed as described in the Methods). Results are given as mean ± SD. *P<0.05 versus control, **P<0.02 versus control, #P<0.05 versus bleomycin + vehicle.

The comparison between the spectral profiles of control and bleomycin-treated lung tissue shows extensive differences in all the spectral range. Most of these differences are statistically significant (S1 Table) and demonstrate dramatic metabolic consequences of bleomycin-induced lung injury. As expected, amino acids closely involved in the formation and structure of collagen, glycine and proline, show the most prominent change with more than two fold increases as a consequence of the development of lung fibrosis in the bleomycin + vehicle group (Fig 3). The animals treated with roflumilast prevented the increase of glycine and proline induced by bleomycin near to control levels (Fig 3). Metabolomic observations were also reproduced in histological experiments. Lung sections of mice treated with bleomycin showed intense tissue fibrosis with collagen deposition quantified by a significant increase of the Ashcroft score. Roflumilast significantly decreased the Ashcroft score, collagen deposition and lung fibrosis in the histological analysis (Fig 3).

**Fig 4:**
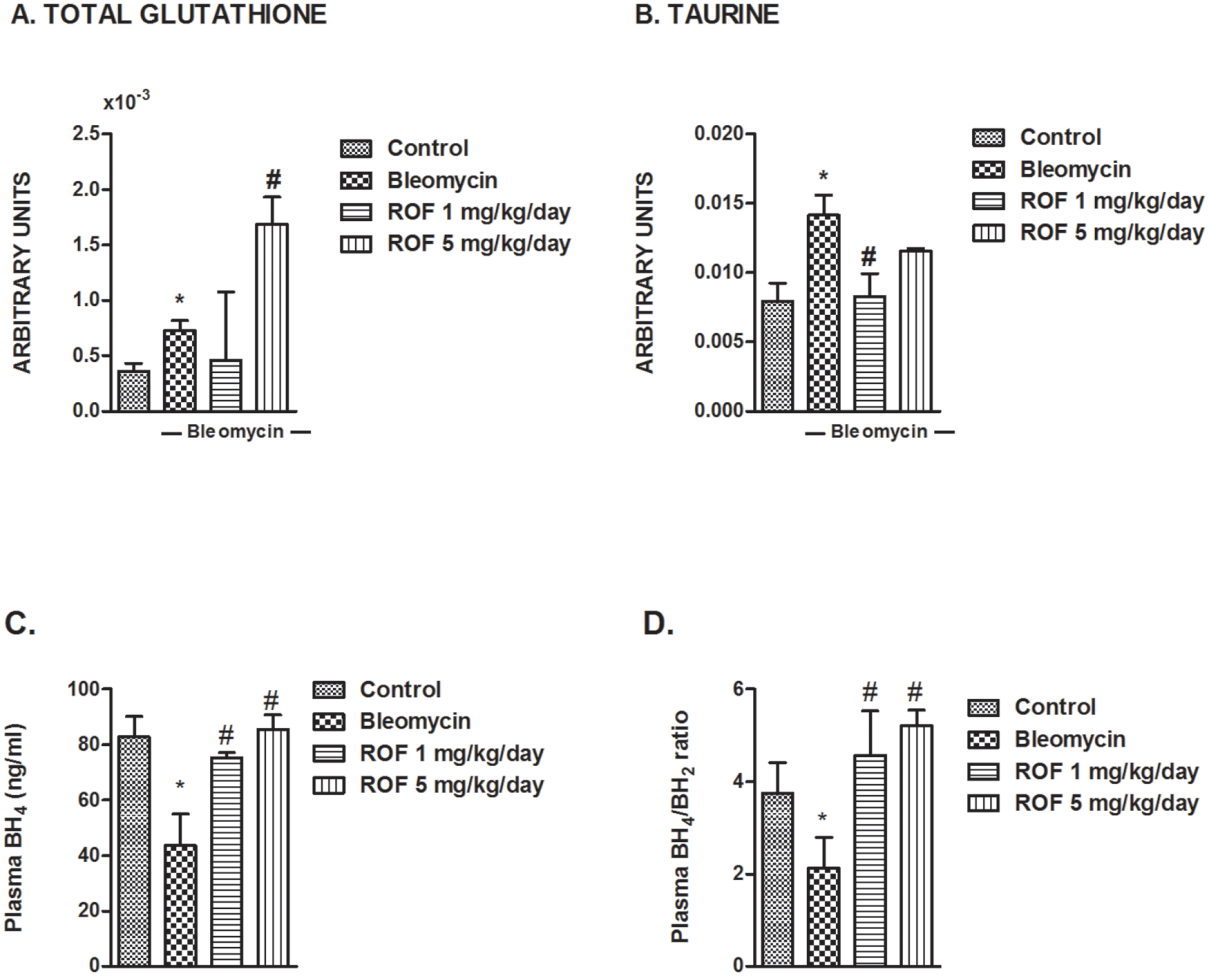
Effects of roflumilast on bleomycin-induced metabolites related to oxidative stress in mouse lungs. Mice received a single dose of bleomycin (3.75 U/kg) intratracheally at day 1 and roflumilast (1 or 5 mg/kg/day p.o., once daily) or vehicle was administered from day 1 to 14 (*preventive protocol*) until analysis at day 14. (A) Total glutathione and (B) taurine were assessed by NMR in murine lung control tissue (n = 17), lung tissue from animals treated with bleomycin + vehicle (n = 14), lung tissue from animals treated with bleomycin + roflumilast at 1 mg/kg/day (n = 8) and lung tissue from animals treated with bleomycin + roflumilast at 5 mg/kg/day (n = 4). Plasma levels of (C) BH4 and (D) BH4/BH2 ratio were measured by high performance liquid chromatography (HPLC). Results are given as mean ± SD. *P<0.05 versus control, **P<0.02 versus control, #P<0.05 versus bleomycin + vehicle.

Oxidative stress plays a key role in lung fibrosis and increases in lungs of bleomycin-challenged mice [25]. Here we found that metabolites protecting against oxidative stress and inflammation, such as total glutathione and taurine showed a marked increase after bleomycin, possibly as a defense mechanism. While bleomycin-induced taurine levels were restored by roflumilast, the total glutathione content in lung tissue was further increased by roflumilast at 5mg/kg/day (Fig 4A and 4B). The latter observation may contribute to mitigate the net burden of oxidative stress in lungs.

Uncoupling of nitric oxide synthase (NOS) secondary to oxidative degradation of BH4 augments peroxynitrite, a powerful oxidant and nitrating agent. We now report a significant decrease in plasma BH4 levels and the BH4/BH2 ratio following bleomycin that was fully restored by roflumilast (Fig 4C and 4D).

**Fig 5:**
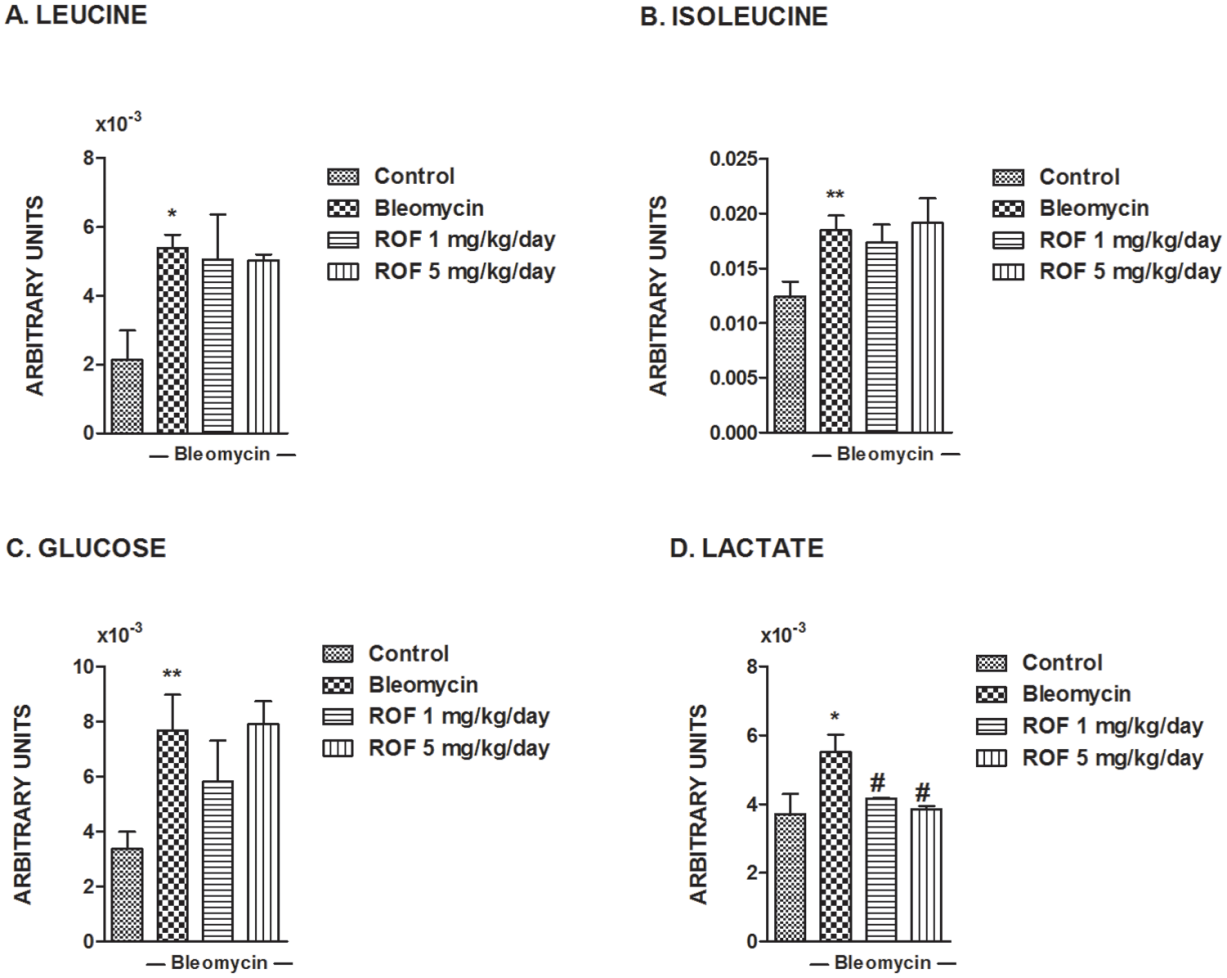
Effects of roflumilast on bleomycin-induced metabolites related to branched chain amino acids leucine and isoleucine, glucose and lactate in mouse lungs. Mice received a single dose of bleomycin (3.75 U/kg) intratracheally at day 1 and roflumilast (1 or 5 mg·kg^-1^·d^-1^ p.o., once daily) or vehicle was administered from day 1 to 14 (*preventive protocol*) until analysis at day 14. Leucine (A), isoleucine (B), glucose (C), and lactate (D) were assessed as described in Methods in murine lung control tissue (n = 17), lung tissue from animals treated with bleomycin + vehicle (n = 14), lung tissue from animals treated with bleomycin + roflumilast 1 mg/kg/day (n = 8) and lung tissue from animals treated with bleomycin + roflumilast 5 mg/kg/day (n = 4). Results are given as mean ± SD *P<0.05 versus control, **P<0.02 versus control, #P<0.05 versus bleomycin + vehicle.

Other amino acids, such as glutamate, alanine, arginine experience a moderate but statistically significant increase following bleomycin (S1 Table). Along the same line, branched chain amino acids leucine, isoleucine and valine show a strong increase under bleomycin suggesting inhibited protein synthesis or increased protein degradation (Fig 5A and 5B, S1 Table). These changes in branched chain amino acids levels were not prevented by roflumilast at either doses suggesting that the PDE4 inhibitor does not affect bleomycin-induced changes in protein synthesis.

Lung glucose content increased following bleomycin along with lactate, suggesting reduced glucose uptake, high glycolytic activity and decreased pH by lactic acid (Fig 5C and 5D). Although roflumilast did not modify glucose tissue concentrations, we observed a significant inhibition of lactate for both roflumilast concentrations (Fig 5C and 5D).

**Fig 6:**
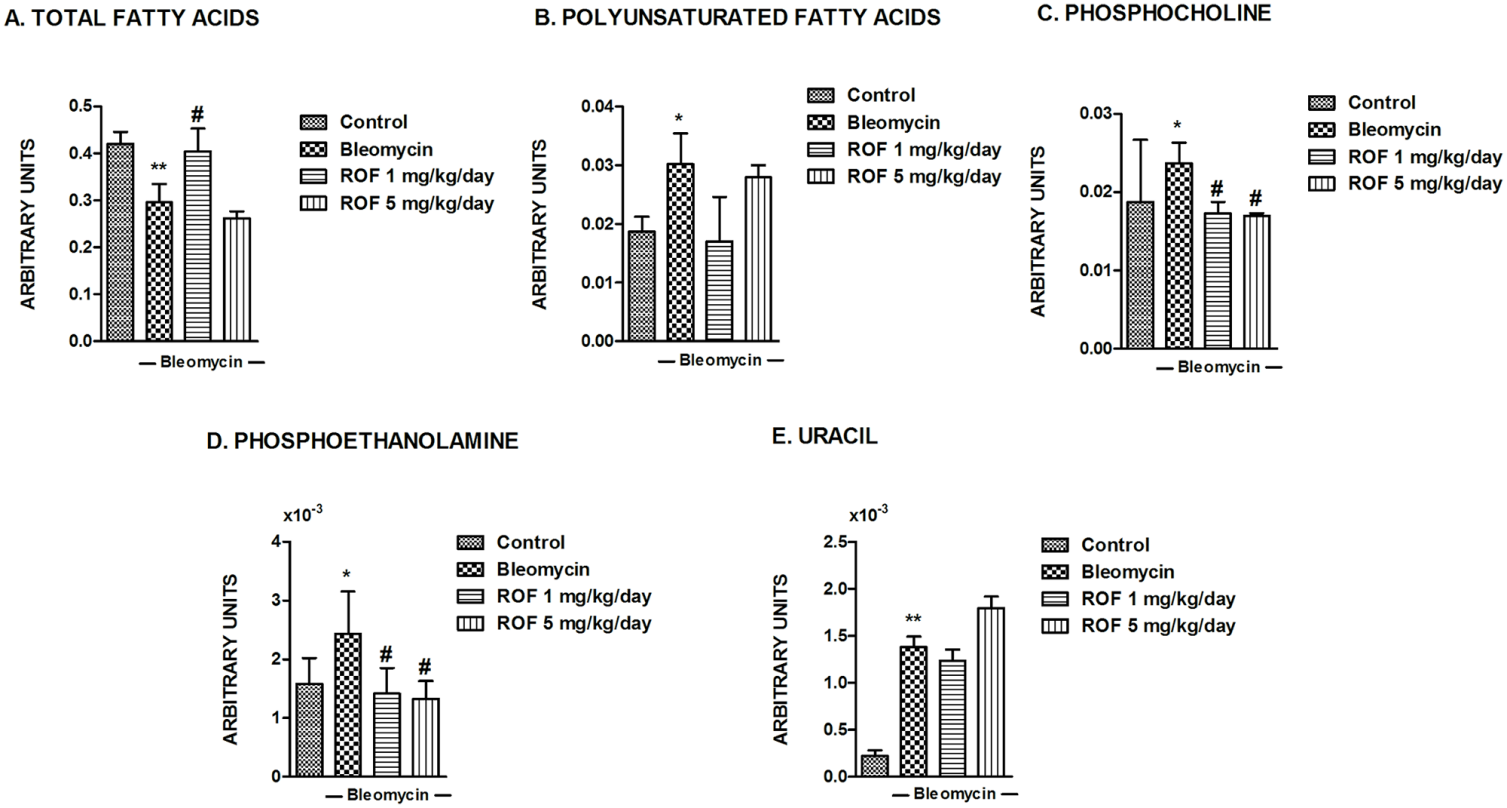
Effects of roflumilast on bleomycin-induced metabolites related to fatty acids, phospholipids and uracil in mouse lungs. Mice received a single dose of bleomycin (3.75 U/kg) intratracheally at day 1 and roflumilast (1 or 5 mg/kg/day p.o., once daily) or vehicle was administered from day 1 to 14 (*preventive protocol*) until analysis at day 14. Total fatty acids (A), polyunsaturated fatty acids (B), phosphocholine (C), phosphoethanolamine (D), and uracil (E) metabolites were assessed as described in Methods in murine lung control tissue (n = 17), lung tissue from animals treated with bleomycin + vehicle (n = 14), lung tissue from animals treated with bleomycin + roflumilast 1 mg/kg/day (n = 8) and lung tissue from animals treated with bleomycin + roflumilast 5 mg/kg/day (n = 4). Results are given as mean ± SD. *P<0.05 versus control, **P<0.02 versus control, #P<0.05 versus bleomycin + vehicle.

Fatty acids pattern also changes following bleomycin with a decrease in the signal belonging to total fatty acids (FA) and an increase in the signal of monounsaturated (MUFA) and polyunsaturated (PUFA) fatty acids (Fig 6A and 6B). The phospholipid precursors, phosphocholine (PC) and phosphoethanolamine (PE) were increased in lungs following bleomycin. High levels of phospholipid precursors may illustrate membrane turnover and cell proliferation. The bleomycin-induced increases in PC and PE were abolished by roflumilast at either doses (Fig 6C and 6D). Finally, uracil increased following bleomycin treatment suggesting that bleomycin changes nucleotide metabolism. This increase was not affected by roflumilast (Fig 6E).

## Discussion

In this study we investigated metabolic changes in the murine model of pulmonary fibrotic remodeling secondary to bleomycin and their potential prevention by roflumilast, a PDE4 inhibitor. We characterized the metabolic fingerprints following bleomycin in the presence or absence of roflumilast in intact lung tissue using HR-MAS. The multivariate analysis shows that in general, roflumilast prevented the global metabolic changes induced by bleomycin in lung tissue. For the majority of the metabolites the PDE4 inhibitor prevented the changes by bleomycin to at most 10% difference with respect to control levels. Metabolites directly related to DNA damage (probably nucleotide metabolism and protein synthesis) were not affected by roflumilast suggesting that the PDE4 inhibitor primarily acts to mitigate the inflammatory and fibrotic effects of bleomycin.

During the last decades, the knowledge about mechanisms underlying lung fibrotic remodeling has vastly increased. However, although current treatments with pirfenidone or nintedanib have improved lung function and survival, pulmonary fibrosis is still a fatal disease. The bleomycin rodent model is widely used in the assessment of potential anti-fibrotic agents, which may help in designing new patient management strategies [26]. In rodents, bleomycin-induced lung injury begins with (alveolar) epithelial cell injury, followed by an acute inflammatory response and then fibroblast activation resulting in their proliferation and enhanced deposition of extracellular matrix including collagen. Most of the initial damage occurs secondary to bleomycin-induced DNA injury and a rise in reactive oxygen species (ROS) [27]. However, given the strong inflammatory and later fibrotic response bleomycin is likely to produce a variety of metabolic changes in lung cells beyond those related to the DNA injury. We found increases induced by bleomycin in the levels of glycine and proline, amino acids directly related to the synthesis of collagen. The increases of both metabolites were reversed by roflumilast to control levels which translated into reduced lung tissue collagen deposition and fibrotic scores as shown in this and our previous study [10]. These findings reiterate a potentially anti-fibrotic role for roflumilast in addition to its well-known ability to mitigate inflammation [10].

Among the different mechanisms implicated in lung fibrotic remodeling the increase of radical oxygen species (ROS) appears to play a key role. In fact ROS may contribute to repetitive injury of alveolar epithelial type II cells, their transformation into mesenchymal cells (EMT) and apoptosis, transition of fibroblasts into the aggressive, apoptosis resistant and invasive myofibroblast described in IPF, endothelial dysfunction among others [28]. Therefore a treatment that reduces both the generation and enhances the inactivation of ROS may reveal favorable to mitigate lung fibrotic remodeling. In this work we observed that roflumilast dose-dependently increased the lung tissue levels of total glutathione and plasma levels of BH4 in bleomycin exposed mice. Glutathione is a key endogenous antioxidant protecting cells from free radicals. In fact, TGFβ1 mediates some of its pro-fibrotic actions by reducing cellular glutathione levels [29]. The increase of lung glutathione levels after bleomycin intratracheal instillation can likely be explained by a mechanism of defense of lung tissue against the rise in oxidative stress. However, increased glutathione is not sufficient to overcome the increase of ROS in lung tissue [30]. In this regard, we and others showed that in different models relevant to pulmonary fibrosis roflumilast and its active metabolite roflumilast N-oxide reduce ROS generation in numerous types of lung cells and lung tissue [12,31,32]. Specifically, it has been shown that roflumilast inhibits the NADPH oxidase (NOX) activity by suppressing the interaction of rac1 with NADPH oxidase isoenzymes [31]. Furthermore, roflumilast was able to reduce an enhanced expression of NADPH oxidase isoenzymes NOX1, NOX2 DUOX2 and NOX4 secondary to different stimuli in bronchial epithelial cells and lung fibroblast [11,32] which may contribute to the anti-fibrotic profile of roflumilast *in vitro* and *in vivo*. In fact, recent evidence indicates that increased NOX4 expression is involved in lung fibrotic remodeling in idiopathic pulmonary fibrosis [33].

Further we found that roflumilast fully prevented the bleomycin-induced loss in plasma BH4. The co-factor BH4 is required for NO generation by NOS. Reduced BH4 availability as in conditions of enhanced oxidative stress exemplified by IPF entails uncoupling of NOS. Uncoupled NOS generates superoxide radicals aside from NO that together react to peroxynitrite, which contributes to endothelial dysfunction. In this regard we previously observed that BH4 plasma and lung tissue levels are significantly reduced in IPF patients and that targeting the BH4 synthesis 'salvage pathway' with sepiapterin improves pulmonary fibrosis and the characteristic pulmonary vascular remodeling associated [34]. One may postulate that restored plasma BH4 by roflumilast supports the improvement of bleomycin-induced pulmonary vascular remodeling previously described by our group with this PDE4 inhibitor [10].

Phosphocholine (PC) and phosphoethanolamine (PE) are important metabolites in the synthesis and degradation of phospholipids, which are essential components of cellular membranes. We observed changes in the levels of these metabolites in lungs of mice exposed to bleomycin. The interpretation of variations in these PC and PE signals is not trivial. Altered intensity of these signals may not only suggest altered synthesis or degradation of membranes, but it may also indicate changes in the synthesis and degradation combined or just in the mobility of the choline-containing phospholipid parts. In terms of fibrotic remodeling the observed increases in PE and PC signals could be related to lung fibroblast proliferation. In this latter context it is an interesting finding that roflumilast reversed the bleomycin-induced increase of both, PC and PE [35,36].

The levels of branched chain amino acids, common metabolomic markers of protein synthesis and degradation, are increased in lungs suggesting some impairment in the protein turnover following bleomycin. Roflumilast did not influence the bleomycin-induced increases in branched amino acids or uracil in the lungs. Likely, these increases in metabolomic markers related to protein and DNA turnover are not part of the fibrotic process.

Our results indicate that bleomycin indirectly activates extra-mitochondrial anaerobic glycolysis reflected by the increase in lung tissue lactate. The reason for this effect is unclear but it may be related to hypoxic areas within bleomycin-induced fibrotic lesions, as previously described [37,38]. Further, bleomycin might impair mitochondrial functions by facilitating the formation of oxygen radicals. Some of these findings have previously been associated to hepatic fibrosis [39] and probably respond to the scarification of the tissue and the general loss of cell functions associated. Roflumilast reversed the bleomycin-induced increase in lung tissue lactate to control levels. This observation may be the consequence of the ability of the PDE4 inhibitor to mitigate bleomycin-induced oxidative stress, fibrosis and inflammation. Currently, there is scarce information about the metabolomic profile in human pulmonary fibrosis lungs [16,40]. However, one recent study using ^1^H-PASS NMR spectroscopy analysis of lung tissue identified lactic acid as a metabolite significantly increased in tissue from patients with IPF compared with healthy controls [16]. Interestingly, secondary to an up-regulation of LDH5 lactic acid increased and in parallel pH decreased in culture supernatants of TGFß1-stimulated lung myofibroblasts. On the other hand, lactic acid resulting in reduced pH triggers fibroblast to myofibroblast transition attributed to an activation of latent TGFß1. Thus, the mutual interaction between TGFß1 to enhance lactate that activates TGFß1 may constitute a self-perpetuating cycle promoting fibrosis. In this context the novel observation from this study that roflumilast abolished the bleomycin-induced increase in lung lactate may herald a complementary anti-fibrotic mechanism of the PDE4 inhibitor. Future investigations may explore whether the PDE4 inhibitor interrupts the self-perpetuating cycle between TGFß1 and lactate in lung myofibroblasts.

Decrease of pH in lung tissue has been proposed as a risk factor to develop lung fibrosis. In fact gastroesopheagal reflux (GER) has been considered as a risk factor for IPF progression due to chronic microaspirations of gastric content. As a corollary the use of medication to combat GER was described as an independent predictor for longer survival time in IPF and a lower high resolution computed tomography (HRCT) fibrosis score [41]. However these data are from retrospective analyses and would need to be corroborated in appropriate prospective clinical trials. Although, data generated in this work may be considered of potential value to explain anti-fibrotic properties of roflumilast, some limitations have to be highlighted. Thus for example, roflumilast was administered in a *preventive protocol* to be analyzed in both inflammatory and fibrotic processes of the bleomycin animal model. Although we have observed that roflumilast is effective reversing lung fibrosis in a *therapeutic protocol* [10], it would be interesting to analyze the metabolomic profile of roflumilast once lung fibrosis is established. In addition we did not explored metabolomic profile in animals treated only with roflumilast which could be useful to explain systemic effects of roflumilast at baseline.

In summary, bleomycin induced strong metabolic changes in murine lung tissue which suggest increased collagen synthesis (glycine and proline), alterations in oxidative equilibrium (glutathione), a strong inflammatory response (taurine), increased anaerobic glycolytic activity (lactate) and decreased DNA function and protein synthesis (uracil and branched chain amino acids). The prevention of most of these effects by roflumilast supports its suggested anti-inflammatory effects, its ability to inhibit fibrosis and oxidative stress. The findings reported here are in good agreement with previous ‘*in vivo*’ studies on the effects of roflumilast.

## Supporting Information

S1 Table Levels (ppm) of metabolites in control group of mice (saline + vehicle), Bleomycin group (bleomycin + vehicle), Rof 1 group (bleomycin + roflumilast 1 mg/kg/day) and the Rof5 group (bleomycin + roflumilast 5 mg/kg/day) with p-values for comparisons showing the effect of the different treatments. (DOCX)
