# Supplementary material for: Roflumilast Prevents the Metabolic Effects of Bleomycin-Induced Fibrosis in a Murine Model
Source: PLoS One. 2015 Jul 20;10(7):e0133453. doi: 10.1371/journal.pone.0133453 (PMC4507994; doi:10.1371/journal.pone.0133453)
Supplement: S1 Table — (DOCX) [file pone.0133453.s001.docx]

**S1 table:** Levels (ppm) of metabolites in control group of mice (saline + vehicle), Bleomycin group (bleomycin + vehicle), Rof 1 group (bleomycin + roflumilast 1 mg/kg/day) and the Rof5 group (bleomycin + roflumilast 5 mg/kg/day) with p-values for comparisons showing the effect of the different treatments.

|  |  | 1 |  | 2 |  | 3 |  | 4 |  |  |  | **t test (p values)** |  |
| --- | --- | --- | --- | --- | --- | --- | --- | --- | --- | --- | --- | --- | --- |
| **Metabolite** | ppm | Control Mean | Control SD | Bleomycin Mean | Bleomycin SD | Rof 1mg/kg/day Mean | Rof 1mg/kg/day SD | Rof 5mg/kg/day Mean | Rof 5mg/kg/day SD |  | 1 vs 2 | 2 vs 3 | 2 vs 4 |
| Cholesterol (VLDL) | 0.65 | 0.0512 | 0.00546 | 0.0415 | 0.00277 | 0.0476 | 0.00131 | 0.03683 | 0.00346 |  | 0.000 | 0.493 | 0.338 |
| Leucine | 0.95 | 0.0021 | 0.00085 | 0.0054 | 0.00038 | 0.0051 | 0.00130 | 0.00503 | 0.00016 |  | **0.026** | **0.874** | **0.954** |
| Valine | 0.98 | 0.0005 | 0.00007 | 0.0007 | 0.00016 | 0.0007 | 0.00016 | 0.00089 | 0.00013 |  | 0.002 | 0.639 | 0.651 |
| Isoleucine | 1.01 | 0.0124 | 0.00138 | 0.0185 | 0.00137 | 0.0174 | 0.00163 | 0.01917 | 0.00221 |  | **0.015** | **0.567** | **0.555** |
| Total fatty acids | 1.26 | 0.4201 | 0.02554 | 0.2957 | 0.03939 | 0.4042 | 0.04848 | 0.26177 | 0.01463 |  | **0.001** | **0.045** | **0.307** |
| Alanine | 1.47 | 0.0044 | 0.00106 | 0.0069 | 0.00131 | 0.0118 | 0.00254 | 0.01154 | 0.00206 |  | 0.014 | 0.372 | 0.633 |
| Arginine | 1.7 | 0.0011 | 0.00032 | 0.0018 | 0.00046 | 0.0020 | 0.00022 | 0.00233 | 0.00027 |  | 0.017 | 0.951 | 0.961 |
| Lysine | 1.89 | 0.0001 | 0.00149 | 0.0001 | 0.00002 | 0.0001 | 0.00002 | 0.00014 | 0.00002 |  | 0.114 | 0.561 | 0.652 |
| Glutamine | 2.12 | 0.0058 | 0.00111 | 0.0094 | 0.00366 | 0.0080 | 0.00133 | 0.00991 | 0.00195 |  | 0.093 | 0.551 | 0.925 |
| UFA | 2.23 | 0.0330 | 0.00263 | 0.0411 | 0.00311 | 0.0410 | 0.00412 | 0.04044 | 0.00339 |  | 0.031 | 0.128 | 0.821 |
| Glutamate | 2.34 | 0.0096 | 0.00244 | 0.0114 | 0.00164 | 0.0123 | 0.00344 | 0.01302 | 0.00057 |  | 0.043 | 0.103 | 0.453 |
| GSX | 2.54 | 0.0004 | 0.00007 | 0.0007 | 0.00009 | 0.0005 | 0.00061 | 0.00168 | 0.00025 |  | **0.022** | **0.036** | **0.488** |
| Aspartate | 2.71 | 0.0058 | 0.00234 | 0.0096 | 0.00332 | 0.0094 | 0.00368 | 0.00986 | 0.00125 |  | 0.420 | 0.194 | 0.307 |
| Asparagine | 2.94 | 0.0004 | 0.00014 | 0.0008 | 0.00014 | 0.0018 | 0.00006 | 0.00215 | 0.00033 |  | 0.005 | 0.207 | 0.567 |
| Creatine | 3.02 | 0.0016 | 0.00015 | 0.0011 | 0.00013 | 0.0025 | 0.00089 | 0.00333 | 0.00027 |  | 0.032 | 0.041 | 0.184 |
| Choline | 3.2 | 0.0104 | 0.00148 | 0.0149 | 0.00056 | 0.0134 | 0.00134 | 0.01366 | 0.00047 |  | **0.048** | **0.460** | **0.428** |
| Phosphocholine | 3.22 | 0.0187 | 0.00795 | 0.0237 | 0.00260 | 0.0173 | 0.00147 | 0.01697 | 0.00034 |  | **0.045** | **0.035** | **0.038** |
| Proline | 3.41 | 0.0014 | 0.00087 | 0.0033 | 0.00021 | 0.0019 | 0.00034 | 0.00181 | 0.00069 |  | **0.043** | **0.048** | **0.048** |
| Taurine | 3.42 | 0.0079 | 0.00131 | 0.0141 | 0.00146 | 0.0083 | 0.00168 | 0.01157 | 0.00016 |  | **0.016** | **0.049** | **0.065** |
| Myoinositol | 3.54 | 0.0044 | 0.00137 | 0.0091 | 0.00257 | 0.0108 | 0.00324 | 0.01231 | 0.00134 |  | 0.039 | 0.147 | 0.645 |
| Glycerol | 3.56 | 0.0273 | 0.00889 | 0.0444 | 0.01309 | 0.0462 | 0.02027 | 0.04058 | 0.00256 |  | 0.000 | 0.429 | 0.965 |
| Glycine | 3.56 | 0.0028 | 0.00063 | 0.0058 | 0.00122 | 0.0030 | 0.00051 | 0.00341 | 0.00068 |  | **0.043** | **0.039** | **0.041** |
| PEA | 3.98 | 0.0016 | 0.00044 | 0.0024 | 0.00072 | 0.0014 | 0.00044 | 0.00133 | 0.00030 |  | **0.047** | **0.047** | **0.042** |
| Lactate | 4.11 | 0.0037 | 0.00060 | 0.0055 | 0.00052 | 0.0042 | 0.00004 | 0.00385 | 0.00008 |  | **0.032** | **0.049** | **0.042** |
| Glucose | 5.23 | 0.0034 | 0.00061 | 0.0077 | 0.00129 | 0.0058 | 0.00149 | 0.00791 | 0.00082 |  | **0.016** | **0.649** | **0.439** |
| PUFA | 5.33 | 0.0187 | 0.00252 | 0.0302 | 0.00520 | 0.0170 | 0.00751 | 0.02794 | 0.00210 |  | **0.035** | **0.065** | **0.427** |
| Uracil | 7.54 | 0.0002 | 0.00006 | 0.0014 | 0.00011 | 0.0012 | 0.00012 | 0.00179 | 0.00013 |  | **0.000** | **0.156** | **0.464** |
